# Particle Detection System Analysis in the Stratosphere Using High-Altitude Platforms Based on a MMPP-2 Model

**DOI:** 10.3390/s25237340

**Published:** 2025-12-02

**Authors:** Mario Eduardo Rivero-Ángeles, Izlian Y. Orea-Flores, Mario Alberto Mendoza-Bárcenas, Iclia Villordo-Jiménez, Edgar Hernan Rosas Espinosa

**Affiliations:** 1 Centro de Investigación en Computación del Instituto Politécnico Nacional (CIC-IPN), Av. Juan de Dios Bátiz S/N, Nueva Industrial Vallejo, Mexico City 07700, Mexico; iorea@ipn.mx (I.Y.O.-F.); erosase2021@cic.ipn.mx (E.H.R.E.); 2 Centro de Desarrollo Aeroespacial del Instituto Politécnico Nacional (CDA-IPN), Bellisario Domínguez 22, Centro Histórico, Mexico City 06010, Mexico; mmendozab@ipn.mx; 3 Unidad Profesional Interdisciplinaria en Ingeniería y Tecnologías Avanzadas del Instituto Politécnico Nacional (UPIITA-IPN), Av. Instituto Politécnico Nacional 2580, La Laguna Ticomán, Mexico City 07340, Mexico; ivillordo@ipn.mx

**Keywords:** space sensors: contaminant detection, MMPP-2, energy consumption

## Abstract

Many space missions using High-Altitude Platforms (HAPs) are designed to measure contaminant particles in the stratosphere. However, there is no previous performance analysis of the sensors installed in the HAP in terms of the energy required by the detection system and the efficiency of the experiment. In this regard, it is not possible to assess the number of measurements that may be taken by the mission and the energy that it will consume in advance. Considering that energy resources are extremely limited in these space missions, especially in HAPs attached to hot-air balloons that effectively provide High-Altitude Platforms (HAPs), where the weight of the payload is of major importance to the success of the mission, a previous analysis is required to account for the feasibility and pertinence of the contaminant detection system. Building on this, we propose a mathematical analysis to determine the energy consumption of the measurement system based on the potential trajectories and the particle density. Also, the analysis provides an estimation of the number of particles that can be detected by the experiment in order to determine the performance of the sensor system. The model is based on an MMPP-2 (Markov Modulated Poisson Process with 2 states) model under exponential distribution assumptions, which provides a basic model that can be easily extended to other distributions in future works.

## 1. Introduction

High-Altitude Platforms (HAPs) have been used for many years to monitor different variables related to ecology and pollution on Earth. However, due to the high altitude of many HAP systems, detection of contaminants in the atmosphere has not been a primary task. In this sense, a special kind of HAP may prove to be useful to detect different particles suspended in the stratosphere at a low distance that can have a negative impact on different ecosystems and human and animal health. Namely, HAPs placed on hot-air balloons travel at lower altitudes compared to Geo-stationary or even Low-orbit HAPs, allowing us to measure contaminants that can directly affect life on Earth.

Detection of contaminants in the atmosphere is crucial to determine how different particles may travel to different parts of the planet, to detect certain regions where particles are more or less prone to remaining in the atmosphere, to determine potential health hazards due to unusual concentration of pollutants, among many others. As such, using some experiments on board HAPs provide an invaluable tool to determine two main parameters: (a) the particle density in different regions, and (b) the morphology and type of particles found in the trajectory of such HAPs.

To calculate both the density and type of particles during the flight trajectory, we propose the use of cameras to capture images of passing particles through the experiment. It is important to note that we propose to make all measurements in-site and not to bring back to Earth any particle for two reasons: first, to avoid increasing the weight of the HAP, and second, to avoid any type of contamination of the experiment that may jeopardize the recovery mission of the HAP after the balloons land.

Building on this, we propose to have an experiment on board the HAP that allows particles to pass by a tube with a photodetector with two functions: first, it captures the particle arrival, which allows us to determine the density of particles in the atmosphere, and second, it indicates to the camera when to take the photograph of the passing particle to identify the morphology and size of such particles. Note that we focus on the detection of solid particles, as opposed to gases or aerosols, which have been studied before.

Since the use of the camera consumes a high amount of energy, it is not feasible to capture all passing particles since the battery may not last for the complete duration of the mission. As such, we propose to allow the camera to function for a predetermined number of measurements and then to turn it off to reduce energy consumption.

To this end, it is imperative to develop a mathematical analysis to determine the energy consumption and the number of particles that can be measured in the mission. Our proposal consists of an MMPP-2 (Markov Modulated Poisson Process with 2 states) to consider different contaminant densities in the HAP’s trajectory. In view of this, we assume that there are two different zones of particle densities.

- High Density: which may occur when the HAP travels near or directly above highly dense human settlements or industrial zones,
- Low Density: which may occur in natural areas far from human activity

Note that in high (low) density zones, we expect to find a high (low) number of photographs entailing a high (low) energy consumption. As such, it is necessary to consider such differences to provide an accurate analytical model. Also, in case more than two different zones are found, the proposed model can be easily extended to account for this case. It is important to notice that we formulate the hypothesis that the HAP may find different contaminant densities according to the human activity below the HAP’s trajectory. However, it is possible that no such different density zones are present, and microplastics are found pervasively in the stratosphere. In our model, we also account for this case.

The rest of this paper is organized as follows: Section 2 presents related works on HAP and microplastic detection in the stratosphere. Then, in Section 3, we provide a detailed description of the microplastic density detection, including some of the most relevant assumptions and limitations of our work. Then, in Section 4, we provide a potential electronic circuit for the particle detection system, followed by the image processing to determine blurred images during the mission in Section 5. After this, Section 6 develops the mathematical analysis considering an MMPP-2 model. In Section 7, we provide some relevant results to prove the relevance of our work, and we finish this work with the main conclusions.

## 2. Related Work

In this section, we briefly discuss some related works in stratospheric HAP and contaminant particle detection.

Atmospheric monitoring by HAPs has been fundamental in the way we explore the world and measure the impact of human activity on various ecosystems on Earth. In particular, atmospheric monitoring by HAPs has had various applications, for example, climate monitoring [1], radiation from space [2], pollution [3], or monitoring specific areas of the Earth to determine water, thermal, or geological changes or to analyze the development of agriculture [4,5]. However, the deployment of one or more HAPs requires specialized hardware, high costs, and very long preparation times [6,7,8,9]. Furthermore, it has been identified that sensing and processing data in HAPs requires special care, and two different strategies have been proposed. One of them is to obtain samples with the HAP to be processed in specialized laboratories. This method requires that parameter samples be securely stored to prevent contamination or damage to the containers. The balloon’s weight can also change as samples are taken, and if this is not taken into account, it can lead to flight instability. Furthermore, the analysis is not performed in real time [10,11,12]. The second is to obtain the sensor data and transmit them to a ground station for processing. This method allows for real-time data processing; however, several problems exist, such as antenna alignment for transmitting information from the balloon to a ground station, link range, flight duration, energy consumption and therefore battery size, as well as the balloon’s payload weight to include all the hardware for data transmission [4,6,8,9,13].

Given all of the above, the use of low-cost HAP balloons, which allow for the monitoring of different types of particles in the stratosphere, has been of great relevance in recent years. For example, in [9], authors proposed a stratospheric balloon to monitor weather, wind, temperature, humidity, and pressure. The values of these parameters are transmitted to a ground base station. In [10], ionizing particles in the stratosphere were monitored using three balloons. For this purpose, the authors used two plastic scintillators that convert the energy from the ionizing particle into a visible light pulse, which is then converted into an electrical signal and stored in an SD memory card. In order to manage the energy of the sensing module, an on/off switch was included to reduce energy consumption. In [13], a balloon is used to acquire meteorological data and transmit them over several kilometers in real time using LoRA technology. In [11], a balloon was used to monitor radiation from space. In [14], a balloon was designed to detect particles with a diameter of less than 1, 2.5, and 10 mm and their density using two optical particle counters. In [12], a balloon was used to measure the amount of PM particles and their concentration with a filter system and collect them with a sampling bag at the maximum height reached by the experiment. During the balloon’s flight, the filters will trap substances such as solid particles and various gases, including pollutants. Once the filters and the sampling bag have been recovered, the samples will be taken to the laboratory, where the analysis will be performed. Additionally, a fundamental part of this type of HAP is its trajectory. As observed in its applications, the trajectory of the HAP is essential for monitoring. As analyzed in [8,15], the balloons do not have a horizontal propulsion system: they ascend or descend only by inflating and deflating to modify their buoyancy, which allows vertical navigation through various wind layers to determine the horizontal direction. This control method minimizes energy consumption and facilitates prolonged flights in the stratosphere [16,17]. What has been proposed in the literature is to estimate or establish the trajectory of this type of HAPs through reinforcement learning techniques [15,18,19,20,21], however, residence times in different areas of the trajectory of these HAPs have not yet been modeled, as proposed in this work. Finally, in terms of particle detection, although in works such as [22], the authors have applied image processing to determine the concentration of particles in large cities, or in [23,24] where computer vision techniques are applied to defog a multispectral satellite image and provide a range of pollution in the region by estimating the percentage of nitrogen oxides, or in [23] where authors defined an algorithm to aligned the cameras inside a satellite; this type of analysis has not been performed in the detection of particles in the stratosphere, as it is proposed in this work. In this sense, the use of image processing for sample analysis accelerates the process, allowing for the detection of various polluting particles simultaneously. Additionally, more air samples can be taken, as the number of containers included in the balloon’s load is not limited, and this reduces the HPA’s energy consumption by avoiding long-range transmissions.

## 3. Microplastic Density Detection

As mentioned above, we developed a mathematical analysis to evaluate the performance of an experiment in HAPs to detect and estimate the density and morphology of contaminant particles in the stratosphere. Of particular interest is the detection of microplastics, given that these contaminants have been found in remote zones on Earth and the long decomposition time, which is of particular hazard to the animal and human population.

**Figure 1:**
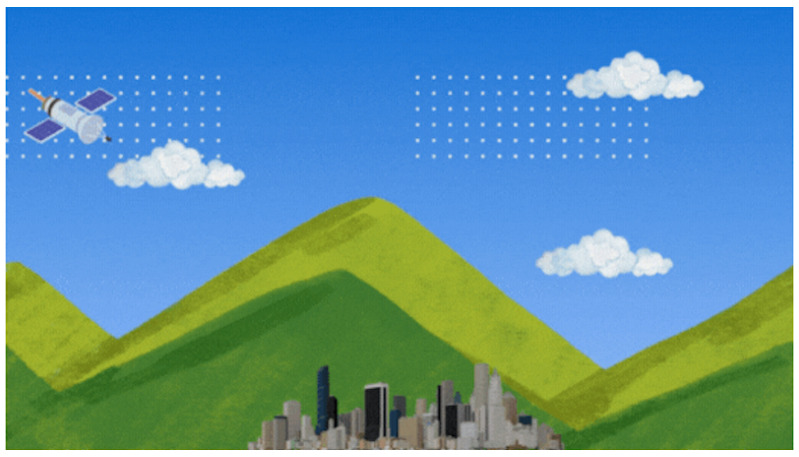
Contaminant particles in the atmosphere encountered by the HAP.

In this regard, the microplastic detection system can be evaluated in terms of energy consumption and detection probability, considering different densities depending on the zones that the HAP is traveling. We assume that the HAP will encounter more contaminant particles in regions with heavy industrial activities or densely populated areas, while in natural zones or rural areas, the contaminant particle density would be low, as shown in Figure 1.

It is important to note that the HAP is traveling through the stratosphere in a certain trajectory, given by atmospheric conditions such as wind, temperature, humidity, etc. Also, the HAP can only capture and detect particles found in this specific trajectory, and it cannot detect particles outside its trajectory. Building on this, it is not possible to know the density in all regions of the stratosphere but only the number of particles per second encountered by the HAP. However, as we now illustrate, the number of particles per second encountered by the HAP gives enough information to estimate the particle density.

**Figure 2:**
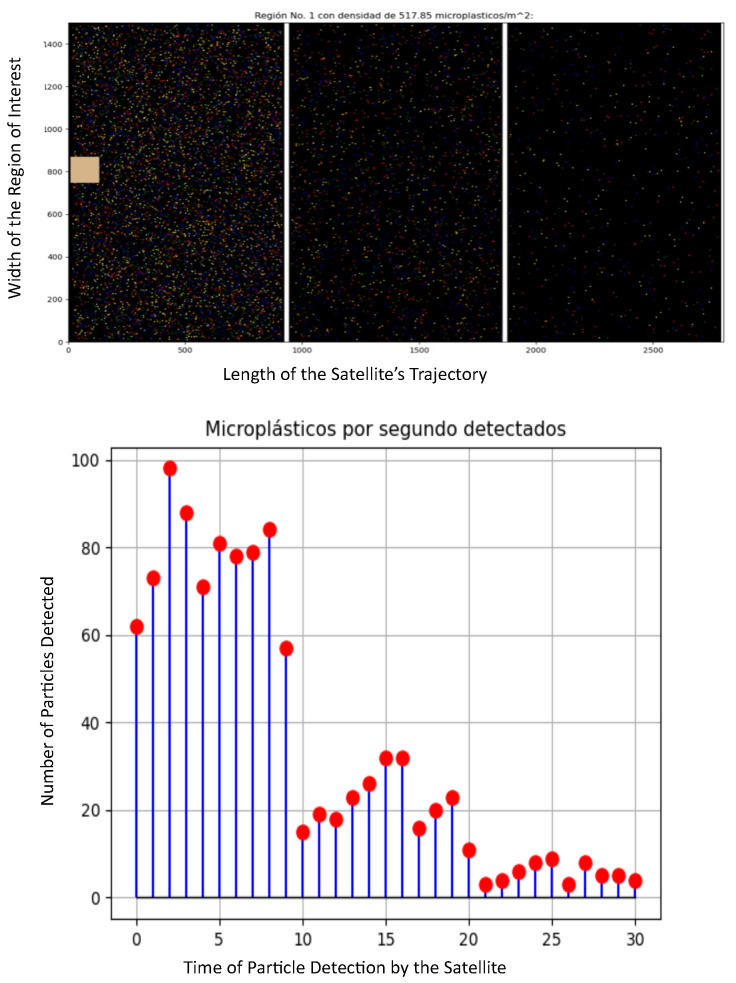
Different particle density in the stratosphere: drastic reduction in the particle’s density.

To prove this, we consider different scenarios where the microplastic density varies and determine if the experiment is valid. These scenarios are only examples used for illustrative purposes. This density is varied in both drastic and mild fashion, and we verify that the number of particles per second in the trajectory closely matches the particle’s density. In all cases, the contaminants are uniformly distributed in the region. In the first scenario shown in Figure 2, we assume the following parameters: Region 1: 518 particles/m^3^, Region 2: 148 particles/m^3^, Region 3: 44 particles/m^3^. The HAP is moving in each zone in a straight line (this assumption is later relaxed to consider random trajectories), and the HAP detected the following conditions: Region 1: 77.2 particles/s, Region 2: 22.38 particles/s, Region 3: 4.7 particles/s. As such, we can see that in this case, the proposed scenario can model the case where the HAP traveled in an industrial zone, and then to an urban zone, and then to a rural zone; the number of particles per second has a high correlation to the contaminant density.

**Figure 3:**
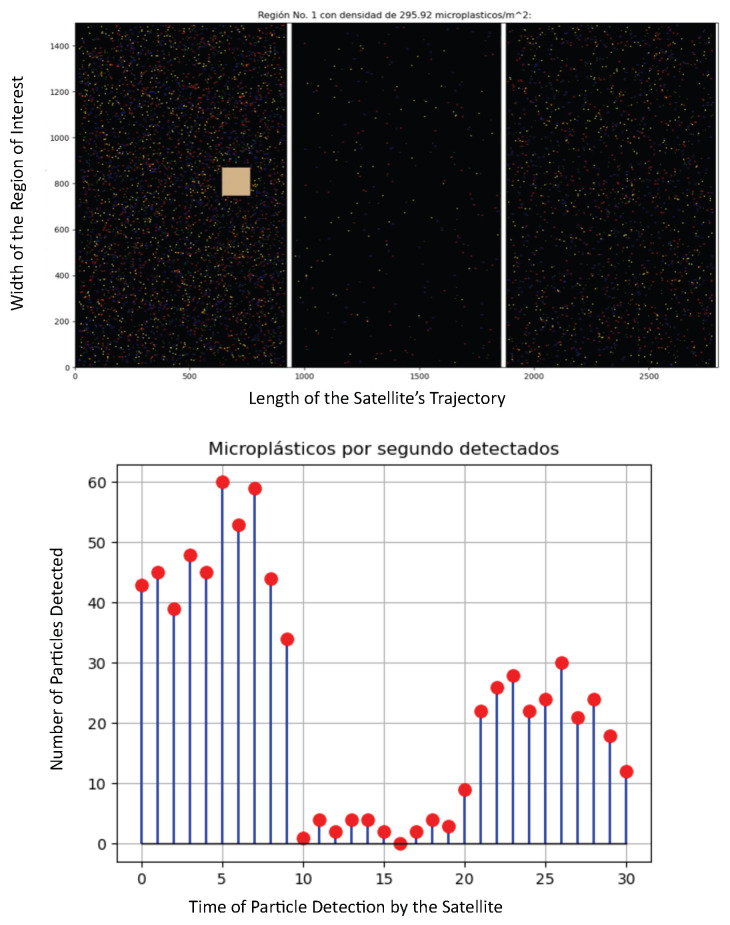
Different particle density in the stratosphere: drastic variation (increase/decrease) in the particle’s density.

In this second scenario presented in Figure 3, we assume the following parameters: Region 1: 295 particles/m^3^, Region 2: 22.1 particles/m^3^, Region 3: 113.16 particles/m^3^. This can be the case where the HAP traveled from an industrial zone into a natural region, such as a forest, a lake, or a desert, and then again to an urban zone. In this case, the HAP detected the following: Region 1: 47 particles/s, Region 2: 2.5 particles/s, Region 3: 21.7 particles/s. Again, the number of particles per second closely matches the particle’s density, and variations are detected almost instantly.

**Figure 4:**
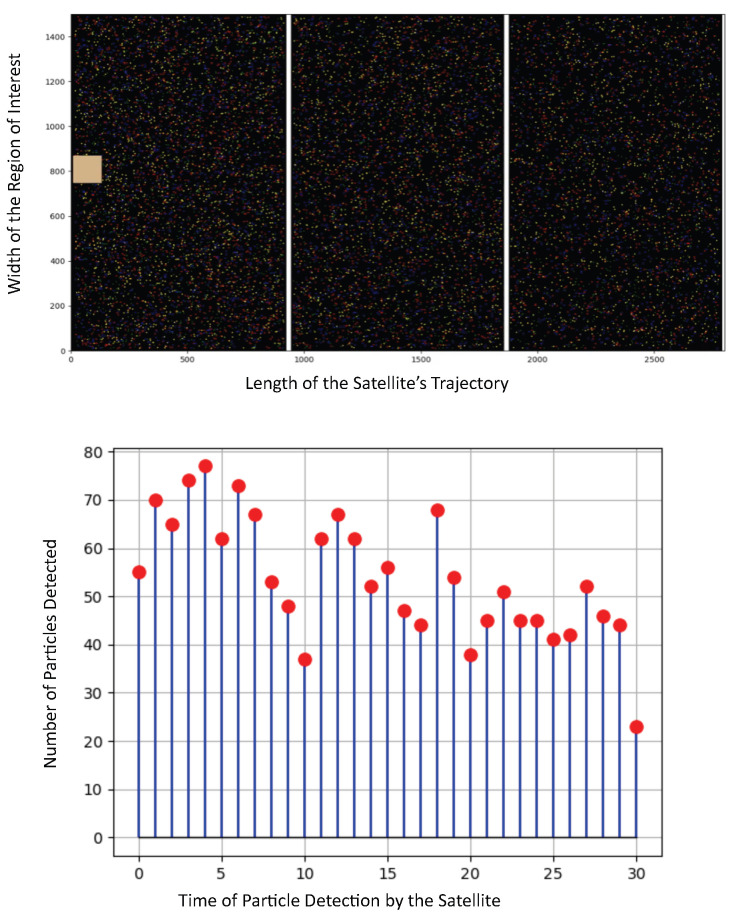
Different particle density in the stratosphere: mild decrease in the particle’s density.

For this last scenario shown in Figure 4, we assume the following parameters: Region 1: 443.87 particles/m^3^, Region 2: 369.9 particles/m^3^, Region 3: 295.92 particles/m^3^. This would be the case of the HAP traveling above a highly populated urban zone into a big suburban zone, where a high contaminant density is also expected, given traffic and commercial regions. Here, the HAP detected the following: Region 1: 61.63 particles/s, Region 2: 55.10 particles/s, Region 3: 43.50 particles/s, which is also highly correlated to the particle’s density.

From these results, we can confidently state that the contaminant density can be estimated only by the number of particles per second. Hence, in the rest of this work, we assume that the HAP can accurately determine the microplastic density only by the number of particles per second that enter the experiment desribed in the following section.

**Figure 5:**
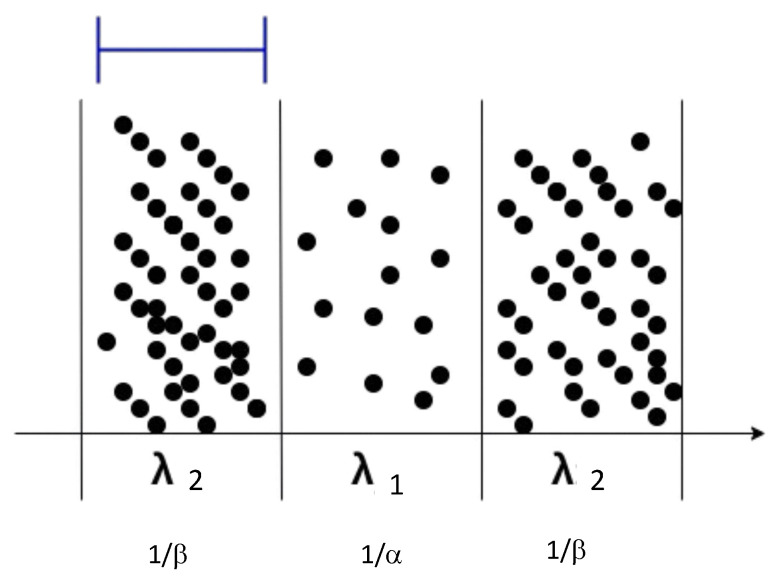
Two particles’ density regions model.

Building on this, we assume that the balloon finds different concentrations of particles in each region, low and high densities of contaminant particles, given by $\lambda_{1}$ and $\lambda_{2}$ partilces/m^3^, respectively. Hence, we only consider two particle densities: high and low, as seen in Figure 5. This is for simplicity reasons, but keep in mind that it can be easily extended to multiple regions in future works.

Now, to accurately evaluate the energy consumption and particle detection capabilities, we no longer consider that the HAP is traveling in a straight line. Instead, we assume that the balloon’s dwelling time is modeled with a random exponentially distributed time with a mean of 1/α and 1/β milliseconds on the low and high density zones, respectively, as shown in Figure 5.

**Figure 6:**
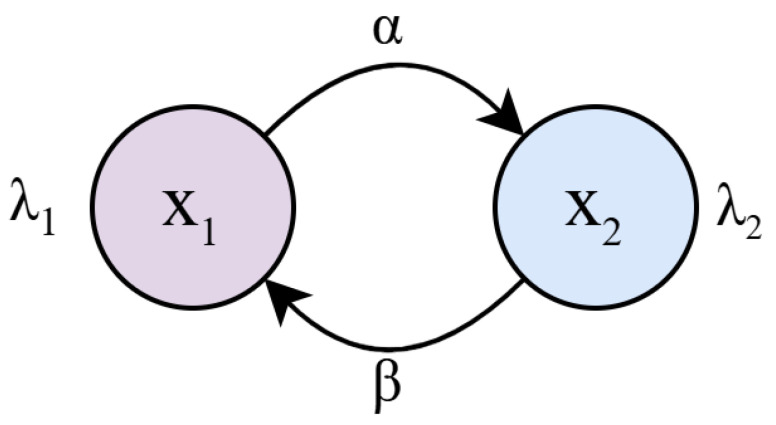
MMPP model that describes the dwelling time of the balloon in different density zones.

The exponential assumption is twofold. For one part, it simplifies the mathematical analysis by allowing the use of Markov chains and the MMPP-2 model. Secondly, it allows an easy extension to non-exponential dwelling times. The MMPP-2 model shown in Figure 6 models the exponential distribution for both the particle density, given by parameters $\lambda_{1}$ and $\lambda_{2}$, and also the dwelling times α and β. Since exponential distributions are considered, they have the memoryless property and then, all the characteristics of Markov chains can be applied to solve this mathematical model.

In view of this, the average particle arrival rate to the HAP is

(1) $$\bar{\lambda }=\lambda_{1}p_{1}+\lambda_{2}p_{2}$$

where

(2) $$p_{1}=\frac{\frac{1}{\alpha }}{\frac{1}{\alpha }+\frac{1}{\beta }}$$

and

(3) $$p_{2}=\frac{\frac{1}{\beta }}{\frac{1}{\alpha }+\frac{1}{\beta }}$$

It is important to remember that the exponential distribution has a Coefficient of Variation (CoV, described in Equation (1)) of 1.

(4) $$CoV=\frac{\sigma }{E[X]}$$

where σ=1/α is the standard deviation and E[X]=1/α is the mean of the exponentially distributed random variable with parameter α, which represents the dwelling time in low-density zones. Similarly, in high density zones, σ=1/β is the standard deviation and E[X]=1/β is the mean time in these conditions.

Hence, if dwelling times in each zone have a CoV value of 1, they can be approximated by this exponential distribution. However, if they have a CoV<1, these times can be approximated by an Erlang distribution. Conversely, if these times have a CoV>1, they can be modeled using a Hyper-Exponential distribution. Both Erlang and Hyper-Exponential are phase-type distributions based on the exponential distributions. Hence, the use of these distributions on the proposed model would only require minor modifications. Therefore, the mathematical analysis developed in this work can be seen as the basic model that can be extended to different conditions and environments in future works.

To the best of our knowledge, there is no data regarding the dwelling times of the HAP in different density zones. Thus, this will also be an important objective of future HAP missions since we believe it will produce important information regarding the contaminant particles’ (such as microplastics) travel patterns and mechanisms of suspension in the atmosphere.

In view of this, the proposed model can be used to evaluate the energy required by the experiment since a camera is assumed to be used to determine the morphology and size of the detected particles by taking images of the passing contaminants. In this regard, the constant use of the camera would rapidly drain the HAP’s energy. Hence, we propose to turn off the camera after *S* sampled particles have been registered.

### Parameter Estimation

This proposal and the consequential model are not based on experimental data, but rather, they represent the first attempt to quantify energy consumption and running time for a future experiment onboard the HAP. As such, we do not have flight data available for use in validating our proposal. Furthermore, based on the performance and results obtained in a future mission, we will be able to validate and adjust this model to extend it, ensuring it accurately reflects practical conditions. In this regard, for our proposal, we consider general parameters and make the assumption of exponential distributions as the basis for future and more precise dwelling times and particle densities. Specifically, the exponential distribution, with a CoV=1, can be easily extended to different distributions with CoV<1 (using the Erlang distribution) and CoV>1 (using the Hyper-Exponential) distribution.

This is the primary reason for us to consider various values for $\lambda_{1}$, $\lambda_{2}$, α, and β. Since we do not have any previous measurements for these parameters, we consider a wide range of potential values that could be encountered in future missions. Hence, in the next mission, we plan to use a timestamp for each particle entering the experiment. Unfortunately, the experiment will remain operational throughout the entire mission (or until the energy is depleted), allowing us to obtain as much information as possible about this arrival process.

However, in future missions, these parameters can be estimated as follows: The particle densities can be determined by the number of particles arriving at the HAP per unit of time. Hence, by simply retrieving the arrival time (timestamp) for each particle entering the experiment, we can easily estimate the particle densities and obtain a figure similar to Figure 5. Then, using these timestamps, we can determine the dwelling times of the HAP in each zone by observing the moments when the particle arrival rate drastically changes.

## 4. Contaminant Particle Detection

In this section, we clearly detail the proposed experiment to detect contaminant solid particles in the stratosphere with two main objectives in mind: First, to know the morphology and size of these potentially harmful contaminants that can easily fall to earth and cause havoc on human and animal health conditions and to the ecosystems in general. Second, to know the particle density in different zones of the globe.

As mentioned before, knowing the particles’ density would give some insight into how contaminants travel long distances (microplastics have been found in very remote zones on Earth); also, it would give guidelines on how many particles should be identified during the HAP mission in order to save energy onboard, allowing other experiments to operate. Specifically, we propose that *S* samples are measured and identified. After detecting this number of particles, the camera and detection system can be turned into sleep mode, effectively saving energy.

**Figure 7:**
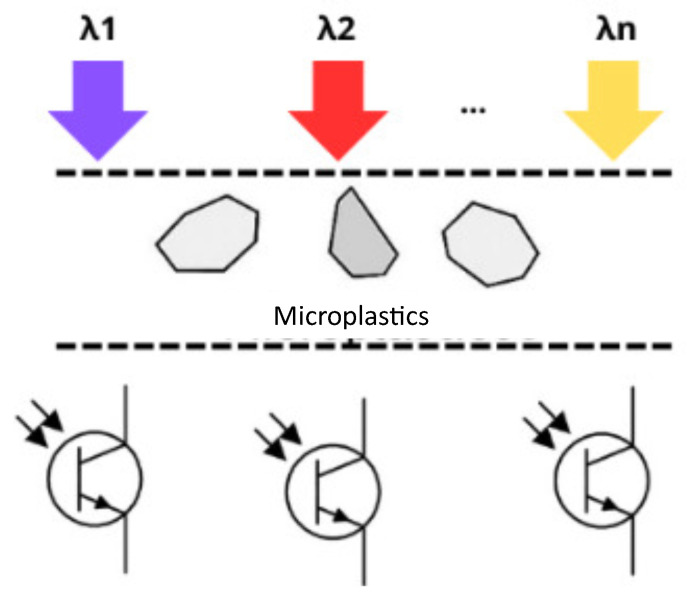
Microplastic detection system.

To count the number of particles that entered the experiment and to have some insights into the chemical composition of the contaminant particles, the payload in the HAP would contain a small aperture where microplastics travel through. The aperture size is determined by the type of contaminant that is being detected. In this aperture, a series of phototransistors are placed, aligned to an LED with a different wavelength, in order to detect different materials. The phototransistors are continuously detecting the light in case there is no contaminant between the LED and the transistor. In case a particle with certain characteristics interrupts the light beam, such that a specific wavelength is blocked or absorbed by the material of the contaminant sample, that particle is then detected by the phototransistor, allowing the type of material through the current conduction and showing a voltage level (VM1), as depicted in Figure 7.

**Figure 8:**
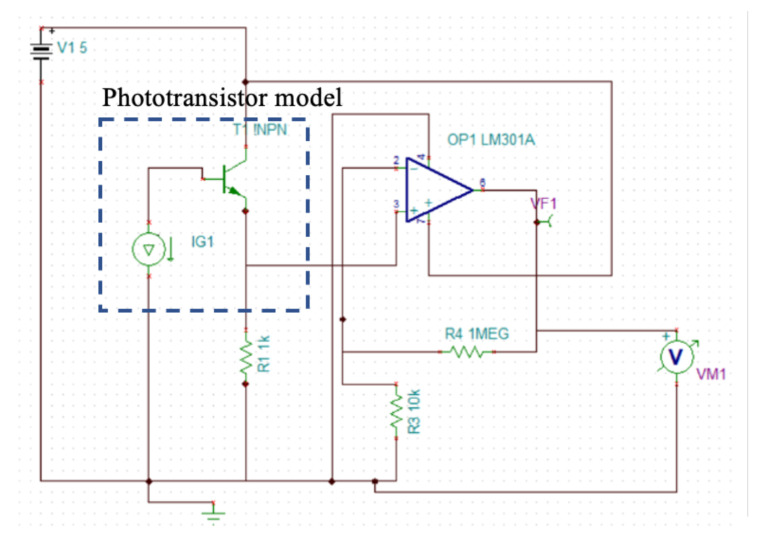
Conditioning and amplification of phototransistor.

**Figure 9:**
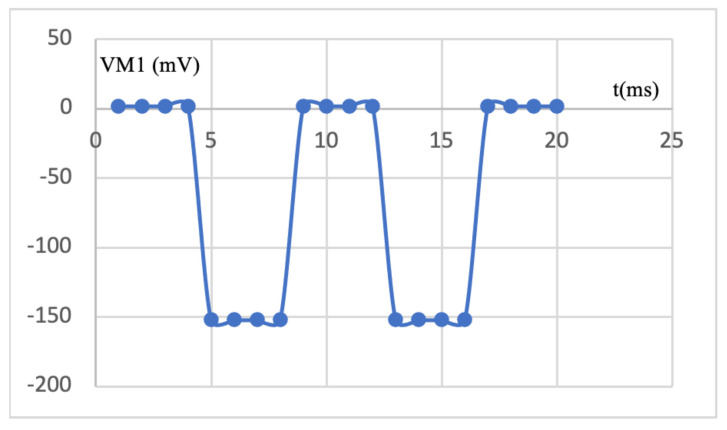
Time Response of the amplification circuit.

Each phototransistor generates a small electric current which is blocked by the passing of a specific type of material. The photodetector is then connected to an amplifier to increase the detected wavelengths, as seen in Figure 8. This electronic circuit operation is shown in Figure 9, where we can clearly observe that when the light accesses the phototransistor, the circuit output changes from 0 to −150 mV.

**Figure 10:**
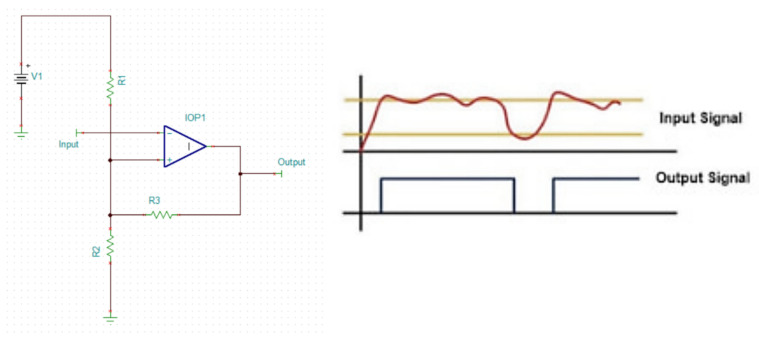
Digitalization of detected particles.

Afterward, we compare the voltage delivered by the amplicator to a specific threshold to determine if there was a particle with specific composition passing by, as seen in Figure 10. This effectively delivers a digital signal that can be easily recorded in any microprocessor onboard.

After this particle detection system, a camera will take a photograph of the passing particle in order to examine the morphology. Note that the use of a camera in these missions has certain challenges. In particular, photographs have to be well focused in order to accurately determine the morphology, and also, each photograph consumes a certain level of energy which, in case the camera is constantly active, would swiftly drain the HAP’s energy. For the former, in the following section, we detail the proposal to determine if the photographs are focused or not. For the latter, we propose to use this digital signal described in Figure 10, entering a simple counter to determine the number of particles that have been detected. Only *S* particles will be detected and photographed in order to save energy. To this end, after this number of particles in each zone has been detected and photographed, the camera will be turned off. We now detail the image processing proposal to detect the focus of the camera.

## 5. Image Processing

**Figure 11:**
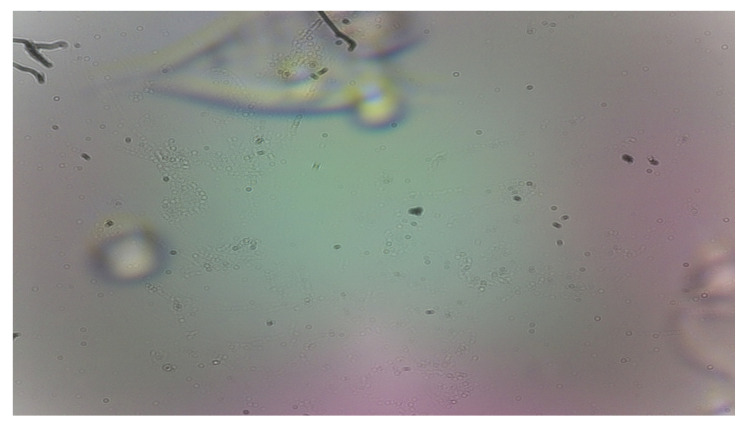
Blured particle photograph taken at the EMIDSS mission.

In this section, we explain in detail the procedure to determine if an image is blurryor out of focus, such as the one depicted in Figure 11.

Recall that in space missions, the camera takes images through a microscope that can be easily de-calibrated due to the drastic temperature changes in the launching procedure and throughout the HAP’s trajectory. In case the microscope loses the original settings, all images taken afterward would be blurry and practically useless. In view of this, we propose an algorithm to detect the level of focus so that the microscope can be recalibrated during the mission and also to stop the image acquisition in case the images are not in focus and reduce energy consumption.

Our proposal is based on the Laplacian Operator of the image, which corresponds to the second derivative of the image as follows:

(5) $$▽=\frac{\delta^{2}}{\delta x^{2}}+\frac{\delta^{2}}{\delta y^{2}}$$

This indicates the variation in the rate of change of the pixels. This operator is useful to identify abrupt changes in the intensity and can be used to detect borders and fine details in the image. Also, it is an isotropic operator, which means that it does not depend on the orientation of the image.

**Figure 12:**
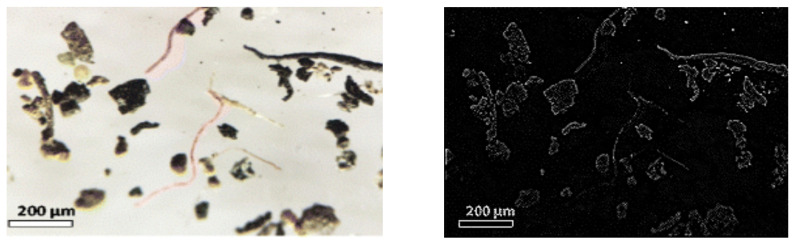
Contour detection for the blurred image.

When the Laplacian Operator is applied, all the regions of the image where abrupt changes are located are enhanced, such as vertical and horizontal borders. Figure 12 presents an example of a microplastic image on the right, and on the left, the same image with the Laplacian Operator is shown.

**Figure 13:**
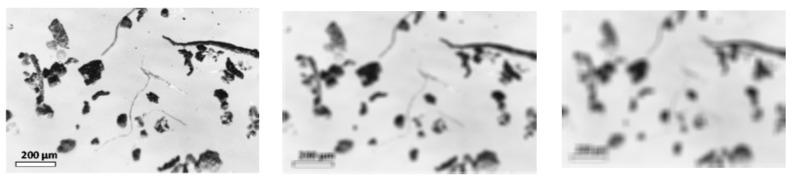
Different levels of unfocused images with variance values: 1886.906, 59.250, and 15.207.

After the Laplacian Operator is applied, we now obtain the variance. A high variance value indicates that there are many intensity changes, which in turn indicates that there are many borders and fine details. This is expected in a focused and clear image. Conversely, in a blurred image, the borders are not well identified, and this entails a low variance. Hence, we use the variance to determine the level of focus (or blurriness) in the images. In Figure 13, we provide the variance to clearly see the relation between the focus level and the variance of the Laplacian operator.

Based on this, the threshold for determining if an image is focused is based on experiments with previous images retrieved by the EMIDSS missions. The variance of the Laplacian Operator is 1886.906 for focused images. As such, we consider that a variance lower than this value corresponds to images with blurred details. In this regard, for future missions where an autofocus mechanism is implemented, the proposal is that if blurred images are detected, the system would attempt to calibrate the microscope to produce clear images. At this point, we have not considered an on-orbit recalibration strategy since it would require a complete mechanical system that could operate independently to correct the microscope mechanism. We believe that this issue falls outside the scope of this work; however, we are certainly interested in future work that would provide a prototype of such a mechanism. Additionally, robustness cannot be measured at this point, but it will be assessed after the following missions.

At this point, if blurry images are detected, the camera can stop taking photos to conserve energy and prevent resource wastage for photos that cannot be used after the mission. This would last for a certain period, allowing future instances to be checked for blurriness by taking new photos. Note that the particle arrival detector can operate independently of the camera, allowing future missions to determine the particle arrival rate and, consequently, the particle density and dwelling times, thereby introducing practical/measured values into the proposed mathematical model.

In view of this, we can conclude that the proposed technique is simple and efficient to identify if the microscope is well calibrated so that it produces focused and clear images that can be used to identify the morphology of the microplastics and other contaminant particles in space missions.

## 6. Mathematical Model

In this section, we provide the main details of the proposed mathematical model. As mentioned above, the mathematical model allows the performance evaluation of the proposed particle detection system in terms of energy consumption and particle detection.

**Figure 14:**
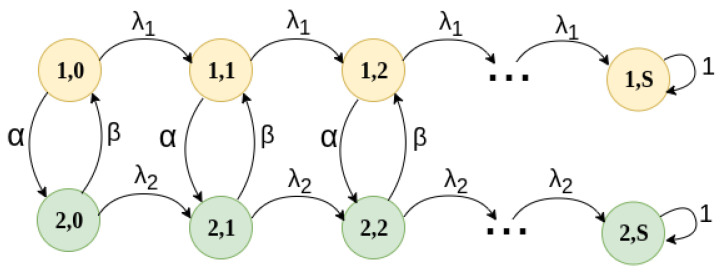
Markov chain for the microplastic detection system.

To this end, we develop a Transitory Continuous Time Markov Chain, illustrated in Figure 14, based on the MMPP-2 model presented in previous sections, where the absorbent state is reached when the maximum number of particles, *S*, is detected.

Building on this, the aforementioned Markov chain has a valid state space $\left(\Omega_{\left(i,j\right)};i=0,1;0\leq j\leq S\right)$ where *i* indicates the density zone: high or low, as determined by the MMPP-2 model presented in Figure 6. Hence, we can see in Figure 14 that the average time in a low-density zone is 1/α; then, the exit rate from a low-density zone is α. Similarly, for high-density zones, the exit rate is β and the average dwelling time in this zone is 1/β. Also, in low-density zones, the rate at which particles enter the measurement experiment is $\lambda_{1}$, while in high-density zones it is $\lambda_{2}$. When the experiment examines *S* particles, it is considered to have enough samples to determine the density and type of particles. As such, the camera and particle detection system can be turned off to save energy resources.

Based on this model, transitions of the Markov chain in state (i,j) for i=0,1 and j<S are as follows:

- To state (i,j+1) with rate $\lambda_{i}$, when a new particle is detected and photographed.
- To state $\left(\bar{i},j+1\right)$ with rate α if i=0 or β if i=1, when the HAP changes region with a different density zone.

In state (i,j) for i=0,1 and j=S, it remains in the same state. Hence, states i,S are absorbing states, and the detection system is turned off, and no more energy or resources are used to this end.

This Markov chain is numerically solved in order to obtain the average absorbing time, $T_{abs}$. Specifically, the average time since the start of the particle detection system, i.e., in state (i,0) for i=0,1, until it reaches state (i,S) for i=0,1.

From this, the time that the system remains in state (1,j), for j=0,1,…,S, is:

(6) $$T_{\left(1,j\right)}=min\left\{T_{\lambda_{1}},T_{\alpha }\right\}$$

where $T_{\lambda_{1}}$ is an exponentially distributed random vaariable with parameter $\lambda_{1}$, and $T_{\alpha }$ is an exponentially distributed random variable with parameter α. Also, the time that the system remains in state (2,j), for j=0,1,…,S, is:

(7) $$T_{\left(2,j\right)}=min\left\{T_{\lambda_{2}},T_{\beta }\right\}$$

where $T_{\lambda_{2}}$ is an exponentially distributed random vaariable with parameter $\lambda_{2}$, and $T_{\beta }$ is an exponentially distributed random variable with parameter β.

Then:

(8) $$T_{abs}=\sum_{j=0}^{S}\sum_{i=1}^{2}T_{\left(i,j\right)}$$

This average absorbing time is directly related to the energy consumed by the experiment, since for each of the *S* particles, the detection system described above in conjunction with the camera consumes $E_{ON}$ energy units. Also, when there are no particles detected, the system is still active, consuming $E_{idle}$ energy units.

As such, the total energy consumed by the particle detection experiment is given by:

(9) $$\bar{E_{Total}}=T_{abs}E_{idle}+SE_{ON}$$

Note that *S* is a design parameter given by the number of samples required to study the problem of contaminants in the atmosphere. Hence, this parameter should be determined by a group of experts in the field of ecology and environmental sciences. On the other hand, the value of $T_{abs}$ is given by the particle density and HAP trajectory. As such, this parameter involves many other variables, such as altitude, wind, humidity, and so on and so forth.

Finally, the values of $E_{idle}$ and $E_{ON}$ are determined by the detection systems and cameras used on board. Therefore, it would have to be determined by direct measurements on the electronic system. In this regard, we propose to evaluate the system performance using normalized theoretical values considering that $E_{ON}>>E_{idle}$, that is, the energy consumed by the camera is much higher than the energy consumed only by the system proposed in Figure 7, Figure 8, Figure 9 and Figure 10, where a light beam is always used to detect passing microplastics. Also consider that the use of the camera involves the use of the focus detection system discussed in the previous section. Building on this, and for practical purposes, we propose to use:

(10) $$E_{ON}=1\;\mathrm{energy}\;\mathrm{units}$$

and

(11) $$E_{idle}=0.0001\;E_{ON}=0.0001\;\mathrm{energy}\;\mathrm{units}$$

Since these are normalized values, when the exact amount of joules is determined for the practical camera and particle detection system (this value is directly calculated by measurement during the mission, considering that temperature changes can have an important impact on the energy consumption), it can be easily scaled to the real energy consumption values. Indeed, the measured amount of joules of the camera system represents 1 energy unit, while the measured energy consumption of the particle detection system is a fraction of energy in relation to $E_{ON}$.

The values of energy consumption in the ON state and idle state, $E_{ON}$ and $E_{idle}$, respectively, consider all possible effects and phenomena related to the active and inactive states, including electronics, camera equipment usage, and transient effects, among others. The reason for this model is that it does not take into account specific equipment or models. Instead, it presents a general energy consumption model that can be easily adapted to the specific characteristics of each experiment. This can be conducted by simply measuring the joules consumed directly from the equipment in both the active mode (ON state) and the inactive mode (idle state). Specifically, each time a particle is detected, a measurement can be performed to estimate the joules consumed in this procedure, and this value corresponds to 1 energy unit of our model, which is why we consider that $E_{ON}=1$ energy unit. Conversely, during the period when no particles are detected, a direct measurement can be made in the experiment to determine the joules consumed during that period. For illustration purposes, we propose that in an inactive state, the experiment consumes 0.0001 times less energy than in the active mode; however, this value can be easily adjusted to account for the specific circuit and equipment used in each case. This model has been utilized in numerous previously published works, demonstrating its efficiency and applicability [25,26,27,28].

## 7. Numerical Results

In this section, we present some relevant results, where we intend to show the average operation time and energy consumption of the proposed particle detection in the stratosphere. Based on these results, the system administrator can determine the appropriate number of particles to detect, *S*, according to the energy budget of the mission. Indeed, a low number of particles detected entails a low energy consumption of the particle sensor and camera activation but may not be statistically sufficient to determine the pollution density and mean morphology of such particles. This is due to the different density zones assumed in the trajectory of the HAP such that if the HAP remains for long times in a high-density zone, it may detect the *S* particles in that region and never take samples in low-density zones or different high-density regions. On the other hand, a high value of *S* results in a sufficiently large number of detected samples, but it would consume more energy, which could affect the performance of the experiment, in the sense that it may not have enough energy to finish the mission or even affect other experiments on board.

To this end, we obtain the average absorption time of the Markov chain depicted in Figure 14, which is related to the number of particles in each density zone. This absorption time determines the average time it takes for *S* particles to be detected. For these results, parameters 1/α and 1/β are measured in milliseconds, while $\lambda_{1}$ and $\lambda_{2}$ are measured in particles per millisecond.

**Figure 15:**
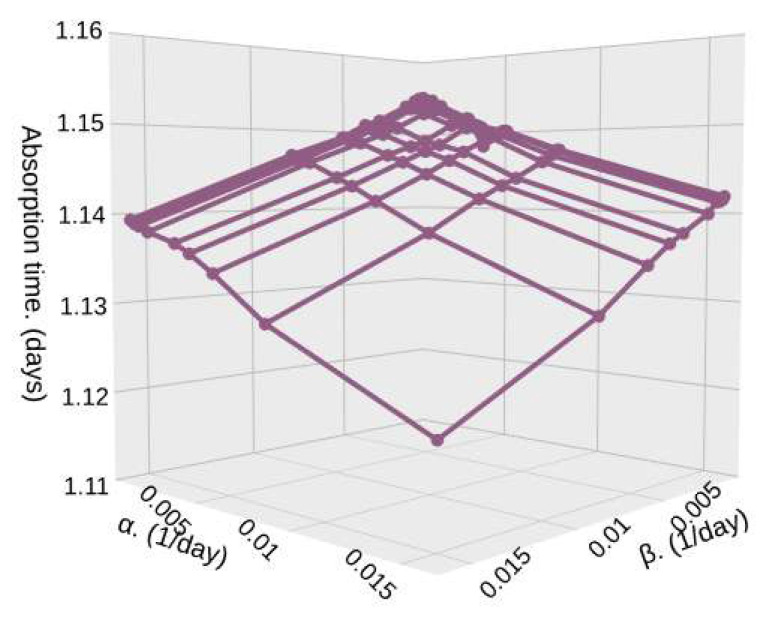
Absorption time for different values of α and β with $\lambda_{1}=0.45$, $\lambda_{2}=0.55$, and S=500.

**Figure 16:**
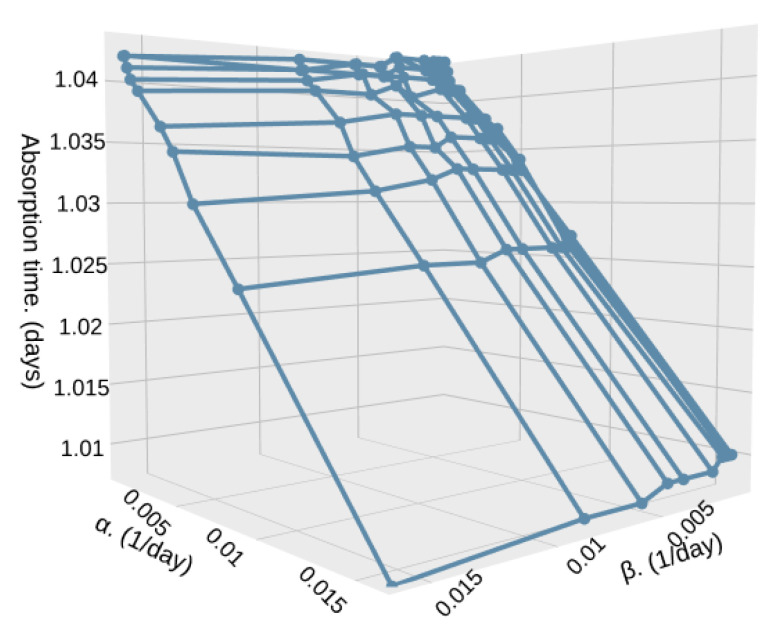
Absorption time for different values of α and β with $\lambda_{1}=0.3$, $\lambda_{2}=0.7$, and S=500.

Figure 15 and Figure 16 shows the absorption time for different densities ($\lambda_{1}$ and $\lambda_{2}$) in high (β) and low (α) particle density zones, and for a relatively low sample size, S=500. In Figure 15, we show a case where the density of both zones is the same or changes in densities are very subtle, such as the one depicted in Figure 4.

In this case, since both zones have a very similar particle density, which means that no matter where the satellite is passing through, it encounters a very similar arrival rate, as shown in Figure 4; then, the values of α and β have little effect on the absorption time. It goes from 100,000,000 milliseconds (1.157 days) to 96,500,000 milliseconds (1.116 days). This small effect happens because α increases, and so the average time in low-density zones 1/α decreases. Hence, the system detects *S* particles faster.

In case the difference in particle density is higher, but still close to each other, $\lambda_{2}=0.7$ and $\lambda_{1}=0.3$, we can see a higher difference in the absorption time, going from 90,000,000 milliseconds (1.041 days) to 87,000,000 milliseconds (1.006 days). The highest impact is on the time that the system remains in low-density zones, while the time in high-density zones is less important. This is because if α increases, i.e., the time in low-density zones (1/α) decreases, the particle detection system would detect more particles since the satellite remains shorter times in low-density zones where the particle arrival rate is low, as shown in Figure 16.

**Figure 17:**
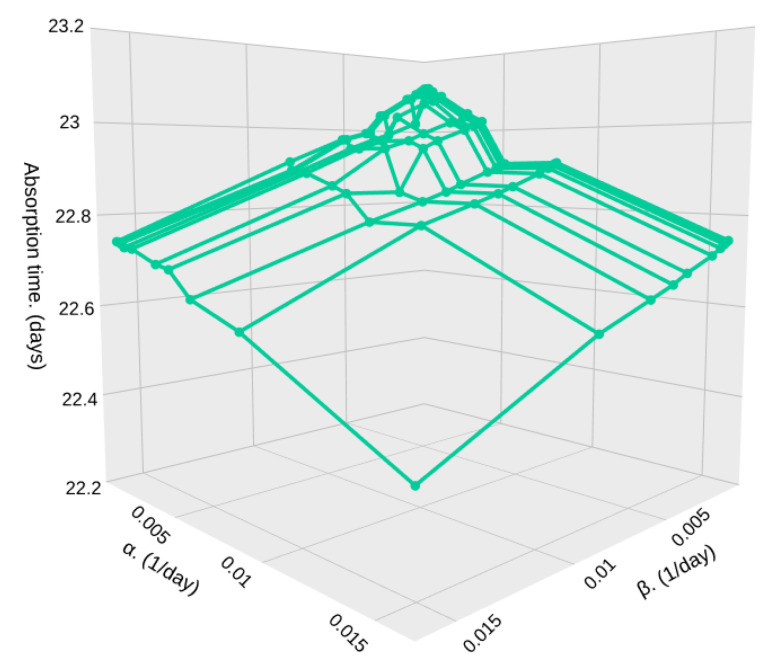
Absorption time for different values of α and β with $\lambda_{1}=0.45$, $\lambda_{2}=0.55$, and *S* = 10,000.

**Figure 18:**
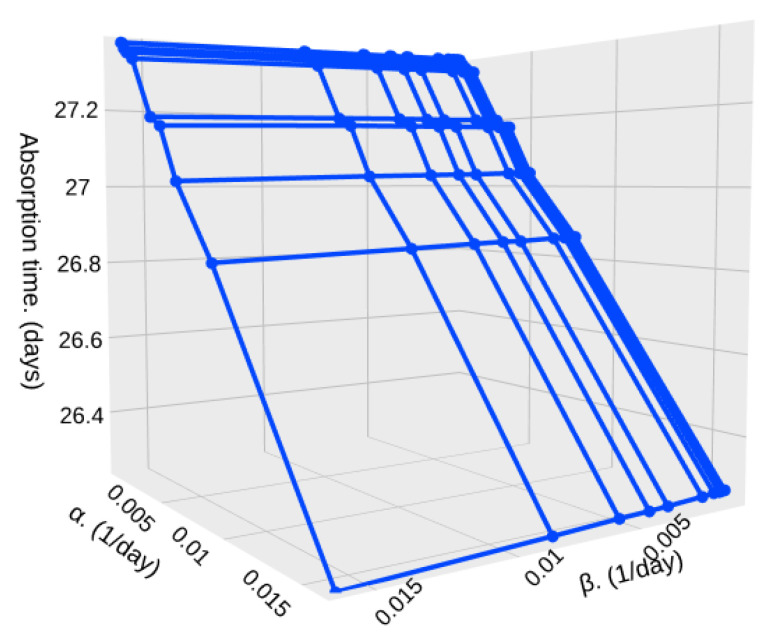
Absorption time for different values of α and β with $\lambda_{1}=0.3$, $\lambda_{2}=0.7$, and *S* = 10,000.

Now, we consider a higher value of *S* = 10,000 sampled particles to stop the particle detection system as seen in Figure 17 and Figure 18. In this case, the effects of α and β are the same as the previous results, but the absorption time is greatly increased, as expected. For the case where particle densities are very similar, the absorption time goes from 2,000,000,000 milliseconds (23.148 days) to 1,930,000,000 milliseconds (22.337 days). Considering that such missions using HAPs only have a duration of a week on average, we can consider this case as a scenario where the particle detection circuit is active during the complete mission duration.

Finally, for *S* = 10,000 sampled particles and a marked difference between particle densities, $\lambda_{1}=0.3$ particles per millisecond and $\lambda_{2}=0.7$ particles per millisecond, we can see that the absorption time goes from 2,360,000,000 milliseconds (27.31 days) to 2,280,000,000 (26.38 days).

From these results, we can also calculate the average energy consumption of the experiment using Equation (9). Recall that the energy consumption values are normalized to the energy consumption of the camera, $E_{ON}$, and the particle presence circuit, which would vary greatly according to the different models and circuit designs. Hence, it is not possible to determine a unique value for the energy consumption. As such, the values presented in Table 1 are only for illustrative purposes. Also, it is possible that energy consumption would vary during the mission compared to the values presented in the data sheets, due to the temperature difference in the stratosphere.

**Figure 19:**
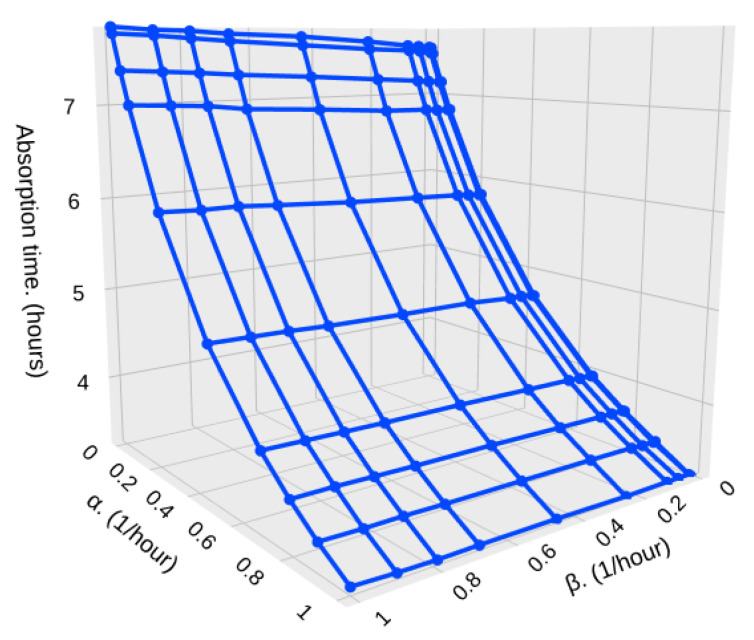
Absorption time for different values of α and β with $\lambda_{1}=1$, $\lambda_{2}=10$, and S=500.

**Figure 20:**
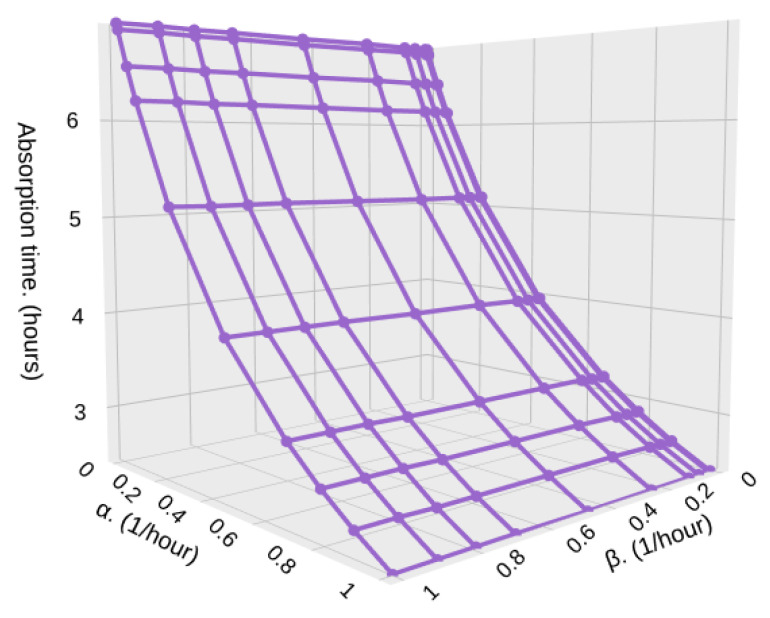
Absorption time for different values of α and β with $\lambda_{1}=1$, $\lambda_{2}=100$, and S=500.

Now, we explore a different scenario, such as the one presented in Figure 3, where there is a marked and high difference in the particle density through the HAP trajectory. Again, we present results for 500 and 10,000 samples. Now, we can observe an impact on the parameter β that was not present when the HAP encounters a very similar arrival rate. We can see in Figure 19 and Figure 20 the cases where 500 samples are taken and there is a difference of one and two orders of magnitude, respectively. We can see that as β increases, the dwelling time in high-density zones decreases and hence, the absorption time increases since it takes longer to complete the 500 samples. Also, the absorption time is lower when $\lambda_{2}$ is higher, since the HAP takes the required samples much faster.

**Figure 21:**
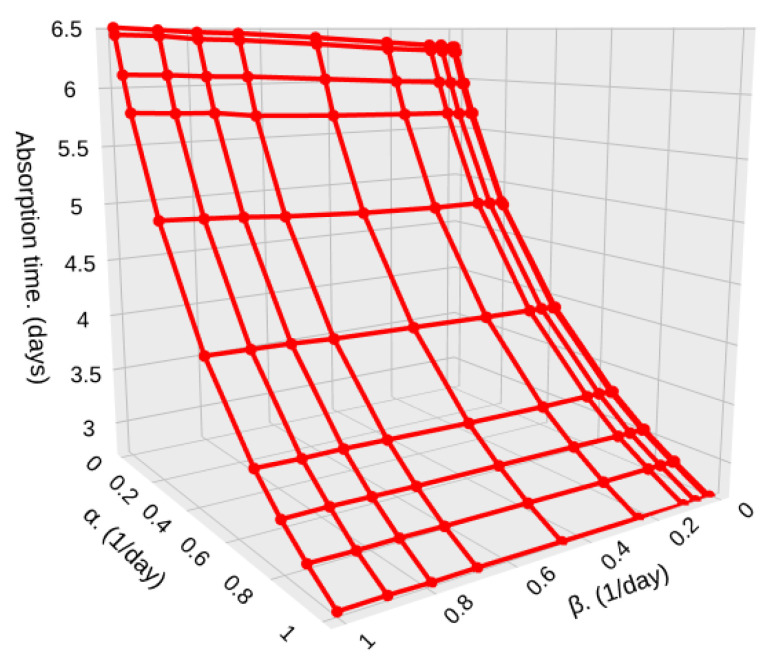
Absorption time for different values of α and β with $\lambda_{1}=1$, $\lambda_{2}=10$, and *S* = 10,000.

**Figure 22:**
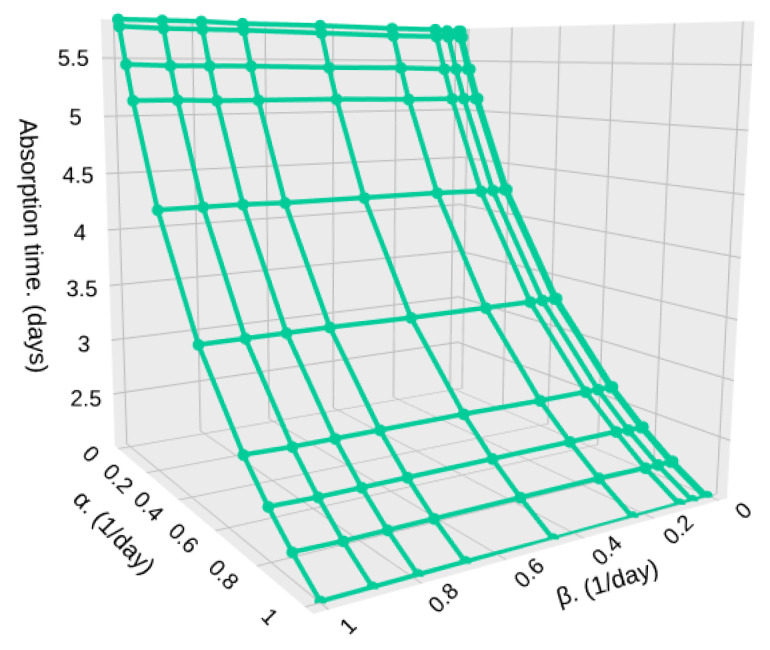
Absorption time for different values of α and β with $\lambda_{1}=1$, $\lambda_{2}=100$, and *S* = 10,000.

Now, in Figure 21 and Figure 22 we present results similar to the previous ones, but for 10,000 samples. We find the same behavior as before, but the absorption is higher, which also entails a higher energy consumption. It is important to note that this absorption time is lower than in Figure 15, Figure 16, Figure 17 and Figure 18, where the particle arrival rate was much lower in the high-density zones simply because when the HAP travels through high-density zones, it captures many more particles than in the previous case. These results highlight the need to have accurate measurements of the microplastic density in the stratosphere, which, to the best of our knowledge, there are not to this day; It would allow for a precise design in terms of energy consumption and mission operation time.

Given these results and adjusting to the specific camera model and the particle detection circuit gives clear guidelines for the battery usage during the mission, which would be most likely shared among all experiments and operational devices on board the HAP. Hence, the results and mathematical model presented in this work allow the project manager to determine the type and capacity of the battery to be used and/or the value of *S* such that enough samples are taken to be statistically representative of the pollution related to microparticles in the stratosphere, and at the same time, that the energy consumption is limited to complete the HAP objectives successfully.

The main objective for considering such a large variation in terms of the absorption time, which ranges from a little over a day to over 20 days, is to allow a detailed design of the experiment since we have two confronted performance parameters, namely, the number of particles to capture and the energy consumption of the experiment. Indeed, for high values of particles to be detected, the higher the energy consumption, the more it reduces the energy available to other experiments on board or increases the battery capacity, weight, and cost. As such, we believe that this mathematical model will allow the experiment design in two possible directions: (1) given a specific battery model (capacity and weight), determine the number of particles (*S*) that can be detected without hindering the mission (allowing enough energy and space to other experiments and electronic systems onboard); (2) given that *S* particles have to be detected (in order to have a statistically significant number of samples), determine the type of battery that has to be used in the mission (given that it complies with the requirements of the HAP).

Building on this, we present results for low-energy consumption scenarios (characterized by a small number of samples and short absorption times) and high-energy consumption scenarios (characterized by a large number of samples and long absorption times). As a result, in this second scenario, the model clearly indicates that the mission duration must exceed 26 days to obtain such a large number of samples. This does not imply that the HAP has to be in flight for 26 days. Instead, we have to interpret these results as follows: If the model determines that the mission must last over the maximum number of days (practical mission duration) to obtain *S* samples, then we should expect that the experiment would be active during the entire mission, and most likely a lower number of samples would be observed.

As such, we are not concerned at this point with the theoretical model’s ability to provide realistic scenarios but rather with proving the model’s applicability and observing its potential for designing this type of experiment. As such, the rationale for providing high mission times is to observe that these performance parameters, most likely, cannot be achieved under these conditions.

## 8. Conclusions

In this work, we have developed a mathematical framework to calculate the average energy consumed to detect *S* particles by a particle detection experiment that uses LEDs and a camera to take photographs only when a particle is detected in order to determine: (a) the particle density in the stratosphere and (b) the particles’ morphology. It is of major importance to know how particles, such as microplastics, are distributed in the stratosphere, and the type of particles found in this region, to determine the level of contamination in different parts of the globe and understand its effects, like health-related issues in different populations of both human and animal sectors. Of particular interest is to know the impact of microplastics on oceans and rivers as well as forests, where major ecological factors can be perturbed by the presence of such a contaminant. This is because the penetration of microplastics in urban and rural areas is well known and relatively easy to measure, but if we can determine how these particles travel through the stratosphere, we can later determine possible solutions to limit plastic usage and wastage to reduce the presence of microplastics in remote areas.

To guarantee that the photographs of the particles passing by the detection circuit are useful in the sense that they are clear enough to be used to study the morphology, we also propose a blur detection algorithm based on the Laplacian Operator to calibrate the equipment or, if this is not possible, to avoid energy wastage by stopping taking photographs that are not clear enough.

The mathematical analysis is based on an MMPP-2 model that considers two density zones in the trajectory of the HAP. At this point, we assume that times in each zone are exponentially distributed random variables since we do not have related data regarding this issue. However, in future works, we intend to relax this assumption by considering other distributions to generalize this analysis. Also, we propose some energy consumption parameters that can be different once the experiment is built and used in a mission to obtain a real and practical value of energy consumption by the camera and electronic circuits during a real mission. Finally, these HAP missions are expected to determine the real and exact values of particle arrival rates, $\lambda_{1}$ and $\lambda_{2}$. As such, in this analysis, we propose hypothetical values assuming that when the HAP is traveling through highly populated and industrialized regions, the particle arrival rate would be higher than in natural and deserted areas. This hypothesis would be confirmed or rejected once a mission measures the particle arrival rate. Hence, these results are illustrative but useful for the mission design.

The results obtained in this work can be used to select the battery model and capacity to provide enough energy to detect *S* contaminant particles that will be statistically relevant for future studies.

## Figures and Tables

**Table 1:** Energy consumption (energy units) of the particle detection experiment under different environments.

*S*	Minimum Value	Maximum Value
	$\lambda_{1}≃\lambda_{2}$	
500	10,150	10,500
	$\lambda_{1}<\lambda_{2}$	
500	9200	9500
	$\lambda_{1}≃\lambda_{2}$	
10,000	203,000	210,000
	$\lambda_{1}<\lambda_{2}$	
10,000	238,000	246,000

## Data Availability

The original contributions presented in this study are included in the article. Further inquiries can be directed to the corresponding author.
